# Challenging the Pleiotropic Effects of Repetitive Transcranial Magnetic Stimulation in Geriatric Depression: A Multimodal Case Series Study

**DOI:** 10.3390/biomedicines11030958

**Published:** 2023-03-21

**Authors:** Vincenzo G. Nicoletti, Francesco Fisicaro, Eugenio Aguglia, Rita Bella, Damiano Calcagno, Mariagiovanna Cantone, Carmen Concerto, Raffaele Ferri, Ludovico Mineo, Giovanni Pennisi, Riccardo Ricceri, Alessandro Rodolico, Giulia Saitta, Giulia Torrisi, Giuseppe Lanza, Manuela Pennisi

**Affiliations:** 1 Department of Biomedical and Biotechnological Sciences, University of Catania, 95123 Catania, Italy; nicovigi@unict.it (V.G.N.); drfrancescofisicaro@gmail.com (F.F.); liberiano@hotmail.it (D.C.); manuela.pennisi@unict.it (M.P.); 2 Psychiatry Unit, Department of Clinical and Experimental Medicine, University of Catania, 95123 Catania, Italy; eugenio.aguglia@unict.it (E.A.); c.concerto@policlinico.unict.it (C.C.); ludwig.mineo@gmail.com (L.M.); alessandro.rodolico@me.com (A.R.); giulia.saitta.91@gmail.com (G.S.); giuliatorrisi2305@gmail.com (G.T.); 3 Department of Medical and Surgical Sciences and Advanced Technologies, University of Catania, 95123 Catania, Italy; rbella@unict.it; 4 Neurology Unit, Policlinico University Hospital “G. Rodolico–San Marco”, 95123 Catania, Italy; m.cantone@policlinico.unict.it; 5 Clinical Neurophysiology Research Unit, Oasi Research Institute-IRCCS, 94018 Troina, Italy; rferri@oasi.en.it (R.F.); pennigi@unict.it (G.P.); 6 Stroke Unit, Neurology Unit, Department of Neuroscience, Ospedale Civile di Baggiovara, Azienda Ospedaliero-Universitaria di Modena, 41126 Modena, Italy; riccardoricceri@gmail.com; 7 Department of Surgery and Medical-Surgical Specialties, University of Catania, 95123 Catania, Italy

**Keywords:** neuromodulation, neural plasticity, mood disorders, neurotrophins, non-pharmacological interventions, cortical excitability, cerebral hemodynamics, case series

## Abstract

Background: Although the antidepressant potential of repetitive transcranial magnetic stimulation (rTMS), the pleiotropic effects in geriatric depression (GD) are poorly investigated. We tested rTMS on depression, cognitive performance, growth/neurotrophic factors, cerebral blood flow (CBF) to transcranial Doppler sonography (TCD), and motor-evoked potentials (MEPs) to TMS in GD. Methods: In this case series study, six drug-resistant subjects (median age 68.0 years) underwent MEPs at baseline and after 3 weeks of 10 Hz rTMS on the left dorsolateral prefrontal cortex. The percentage change of serum nerve growth factor, vascular endothelial growth factor, brain-derived growth factor, insulin-like growth factor-1, and angiogenin was obtained. Assessments were performed at baseline, and at the end of rTMS; psychocognitive tests were also repeated after 1, 3, and 6 months. Results: Chronic cerebrovascular disease was evident in five patients. No adverse/undesirable effect was reported. An improvement in mood was observed after rTMS but not at follow-up. Electrophysiological data to TMS remained unchanged, except for an increase in the right median MEP amplitude. TCD and neurotrophic/growth factors did not change. Conclusions: We were unable to detect a relevant impact of high-frequency rTMS on mood, cognition, cortical microcircuits, neurotrophic/growth factors, and CBF. Cerebrovascular disease and exposure to multiple pharmacological treatments might have contributed.

## 1. Introduction

Depression, one of the main causes of disability worldwide, is associated with a significant impairment of several areas of daily functioning and a poor quality of life, especially in older adults [1]. Furthermore, depression is the most common neuropsychiatric precursor of dementia in late life [2], which ultimately results in a continuously increasing healthcare service request and related economic costs and social burden [3].

Recently, several age-related dysfunctional mechanisms affecting the neurovascular unit have been identified, both in animal models and in patients with neurological and psychiatric diseases, including those with geriatric depression (GD), where the frequent occurrence of chronic subcortical vascular disease (the so-called “vascular depression”) identified an archetypal neuropsychiatric disorder [4]. In this context, recent lines of research focus on the identification of any neuroprotective factors of the neurovascular unit [5]. Among these, neurotrophins (i.e., a family of proteins involved in the development, functioning, and survival of neuronal cells) and angiogenic factors (i.e., a group of molecules, mostly polypeptides, which are crucial in the formation and maintenance of blood vessels) are among the most studied [6,7]. Of note, some growth factors and neurotrophins appear to play a key role in the age-related decline of neurovascular functions and, therefore, they may cooperate in modulating all the neurovascular-based tasks, with effects on mood, cognition, and behavior, among others.

Changes in the serum level of these circulating molecules can be detected and monitored through different biochemical approaches to gain further insights on the pathophysiology of neurological and psychiatric disorders (including GD), to provide additional diagnostic hints, and to evaluate the efficacy of therapeutic interventions. In this context, the neurotrophins and growth factors that seem to be of particular interest are the brain-derived neurotrophic factor (BDNF) [8,9], the vascular endothelial growth factor (VEGF) [10,11], the angiogenin [12], the insulin-like growth factor-1 (IGF-1) [13], the nerve growth factor (NGF) [14], and the endothelin-1 (END-1) [15,16]. As secreting factors from the neurovascular unit, they may have a role in the induction of neurogenesis and the modulation of synaptic plasticity, including complex phenomena (such as “metaplasticity” [17,18]).

In this scenario, the molecular mechanisms of neurovascular dysfunction in GD, and the related risk of dementia, are of utmost importance to disentangle the etiology and designing better clinical interventions for both cognitive and mood disorders in the elderly [19]. In particular, an impairment of the neurovascular coupling has been demonstrated also in the elderly [20], this possibly being a significant contributor to the age-related decline in some cortical functions, such as cognition and mood-affect regulation [21]. Moreover, selective disruption of neurovascular coupling is associated with significant impairment of motor function [22]. The design of new therapeutic interventions (such as new drugs or neuromodulatory protocols) that enhance neurovascular coupling in elderly patients may improve several age-related neuropsychiatric disorders, such as GD.

Up to 40% of depressed patients do not respond adequately to traditional therapeutical approaches, such as one or more medications and psychotherapy [23]. In addition, medication has often partial efficacy, undesired effects and pharmacological interactions are frequent, and ~1/3 of subjects are drug-resistant (i.e., do not reach remission despite ≥2 antidepressant treatments [24]), eventually leading to a chronic depressive disorder. The prevalence of treatment-resistant depression is even higher in patients with GD [25]; therefore, alternative approaches are needed. Among them, non-invasive neuromodulatory therapies are promising, being transcranial magnetic stimulation (TMS) one of the established non-pharmacological interventions for treatment-resistant major depression (MD).

TMS is a safe and painless neurophysiological technique routinely used in clinical practice for the functional evaluation of motor excitability and conductivity along the cortico-spinal tract [26,27,28,29]. Briefly, TMS consists of the delivery of magnetic pulses through the scalp, employing specific stimulation coils, capable of inducing muscle responses that can be recorded with common surface electrodes [30]. The induced motor-evoked potentials (MEPs), recorded by a muscle contralateral to the stimulated cortex, provide information on the anatomic and functional excitation state of several cortical and subcortical areas, the cortico-spinal tract, and intra-/inter-hemispheric connections [31,32,33,34,35,36,37].

The repetitive TMS (rTMS) paradigm, in which trains of magnetic impulses are delivered in rapid succession on the same cortical target, allows to transiently modulate the functioning of the stimulated cortical area, also through the induction/release of neurotrophins and angiogenic factors, the change of electrocortical excitability, and the variation of cerebral blood flow (CBF), thus inducing short- and long-term neuroplastic phenomena [38]. This mainly depends on the stimulation frequency, being high-frequency stimulations (>1 Hz) usually excitatory towards the stimulated cortical target, while low-frequency stimulations (≤1 Hz) are often inhibitory. The mechanisms underlying these neurobiological effects are still partially unknown, though they are hypothesized to be associated with phenomena of long-term depression and long-term potentiation within the brain. As such, rTMS has a wide range of applications in terms of therapeutic and rehabilitative purposes [17,39,40], and in 2008 it received approval by the Food and Drug Administration as an add-on therapy for adult drug-resistant MD [41].

As such, TMS would be appealing for the treatment of GD as well. However, while the antidepressant effect of rTMS in patients with drug-resistant early onset MD is widely recognized, the same cannot be said for GD, especially in those with concomitant chronic subcortical ischemic vascular disease, since the microstructural changes in prefrontal white matter have been recently found to negatively correlate with treatment response [42]. Indeed, although rTMS was found to be effective in the short term in GD with concomitant cerebrovascular pathology, the limited number of studies, variability in study design and protocol, and heterogeneous populations challenge conclusions in this population [43]. Moreover, unlike early onset MD, the impact of rTMS on the neurotrophins and growth factors involved in mood and cognition in GD has not been investigated [44].

In this study, we tested both short- and long-term effects on mood and cognition of high frequency (excitatory) rTMS over the left dorsolateral prefrontal cortex (DLPFC) in GD. As known, DLPFC is a crossroad of several cognitive domains and neuropsychological features, in particular executive functions, behavioral aspects, and mood regulation. DLPFC has also several connections with other areas of the frontal lobe and other brain regions, such as the anterior cingulate cortex (ACC), the insula, the amygdala, and the thalamus, both in the same hemisphere and contralaterally through callosal projections [45]. Moreover, the left DLPFC is the most studied stimulation site of the majority of rTMS trials in depression [46]. Therefore, the non-invasive stimulation of this cortical target can modulate several neural networks and, translationally, exert different clinical effects.

Based on these considerations, here we evaluated the effect of high-frequency rTMS over the left DLPFC on mood, cognition, neurotrophic and growth factors, MEPs to TMS, and CBF to transcranial Doppler sonography (TCD) in a pilot sample of GD patients. We hypothesized that rTMS might exert multimodal effects also in this type of patients.

## 2. Materials and Methods

### 2.1. Participants

In this case series study, a total of six patients with GD (5 males; median age 68.0, interquartile range (IQR) 62.5–78.8; median education: 10.5 years, IQR 5.8–13.0), all right-handed according to the Edinburgh Handedness Inventory [47], were carefully screened and preliminarily assessed for inclusion from the Psychiatry Unit of the Azienda Ospedaliero–Universitaria Policlinico “G. Rodolico-San Marco” of Catania (Italy), from January 2019 to February 2020. An additional seventh participant (a 61-year-old female) was initially enrolled but then excluded since she refused to complete the study. Inclusion criteria were: age ≥ 60 years; diagnosis of MD according to the DSM-5 criteria [48]; clinical onset at or after the age of 60 years; treatment-resistant depression, according to the 2022 Delphi-method-based consensus guideline for definition of treatment-resistant depression for clinical trials [49]. Drug treatment was not stopped or modified (including daily dosages) during the whole procedure. The median duration of the current depressive episode was 8.0 months, with IQR 6.3–9.8.

We considered as exclusion criteria: Mini Mental State Examination (MMSE) [50] < 18 and/or Clinical Dementia Rating scale [51] ≥ 1; other neurological diseases (e.g., stroke, Parkinson’s disease, dementia, multiple sclerosis, traumatic brain injury, epilepsy, etc.) or psychiatric disorders other than depression (e.g., schizophrenia, bipolar disorder, etc.), except for anxiety; severe systemic diseases, acute or chronic severe medical conditions; endocrine diseases, vitamin deficiency (as shown by laboratory exam performed at the entry or by recent exams exhibited by the patients), or exposure to drugs associated with cognitive impairment (e.g., anticholinergics, mood-stabilizers, opiate medications) and/or mood disorders (e.g., steroids; some antihypertensive medications, such as clonidine, guanethidine, methyl-dopa, and reserpine; interferon; L-dopa; retinoic acid derivates; hormonal agents, including corticosteroids, oral contraceptives, gonadotropin releasing hormone agonists, and tamoxifen) [48]; alcohol dependency or substance abuse; any contraindications to magnetic resonance imaging (MRI) and TMS (i.e., implanted devices, previous craniotomy, surgical clips or metal implants, history of epilepsy); and pregnancy.

Informed consent was obtained from all subjects involved in the study, which was conducted in accordance with the Declaration of Helsinki. The Ethics Committee of the Azienda Ospedaliero–Universitaria Policlinico “G. Rodolico-San Marco” of Catania (Italy) approved the study (approval code: N.9/2018/PO). The study fulfilled the CARE checklist for case series investigations (Table S1) [52].

### 2.2. Baseline Assessment

A neuropsychological battery of tests was used to assess cognitive and mood status, both before rTMS (T0) and the day after its conclusion (T1); then, they were repeated at 1 month (T2), 3 months (T3), and 6 months (T4) after the last stimulation session. Tests were administered by the same trained operator (F.F.), who was different from those performing TMS (G.L. and M.P.), in the same lab and experimental conditions.

The tests included: the MMSE as a cognitive screening tool, the Frontal Assessment Battery (FAB) [53] for the global assessment of frontal cognitive abilities, and the Stroop Color–Word Test interference (Stroop T: time, seconds; Stroop E: number of errors; Stroop score: interference score) [54] to assess the ability to inhibit cognitive interference; the Activities of Daily Living (ADL) [55] and the Instrumental Activities of Daily Living (IADL) [56] for the evaluation of functional independence; the 21-item Hamilton Depression Rating Scale (HDRS) [57] and the 30-item Geriatric Depression Scale (GDS) [58] for the quantification of depressive symptoms, and the Apathy Evaluation Scale (AES) [59] for characterizing and quantifying apathy and related symptoms.

A standard 1.5 T-MRI of the brain was performed with T1-, T2-, Fluid Attenuated Inversion Recovery, and proton density-weighted scans; the thickness of each slice was 5 mm, with a slice gap of 0.5 mm. The visual scale of Fazekas was used to grade the severity of ischemic white matter lesions (WMLs), as follows: 0 = absence; 1 = punctuate foci; 2 = beginning confluence of foci; 3 = large confluent areas [60].

A standard electroencephalography (EEG) was recorded before the recruitment. Albeit EEG does not exclude seizure, none suffered from epilepsy, had a history of seizures, or was on any anticonvulsant drug. Peripheral or radicular nerve disease was excluded by a conventional conduction study of the ulnar nerve, bilaterally, including the F-waves.

### 2.3. Laboratory Investigations

Peripheral venous blood was sampled from all participants before rTMS (T0), and a new sample was collected the day following the end of the procedure (T1). Samples were stored in a 5 mL vacuum tube, containing anticoagulant; subsequently, they were centrifuged at 2000 c and at the temperature of 4 °C for 10 min, no later than two hours from the sampling. The obtained sera were stored at −70 °C. The dosage of the biomarkers of interest was processed by using Western Blotting and Immunoprecipitation kits. Percentage level variations of BDNF, NGF, VEGF, IGF-1, ANG, and END-1 were obtained with respect to the baseline (T0).

In Western blotting analyses, to avoid any interference during the electrophoretic protein separation by the overwhelming presence of serum albumin, the serum samples were diluted in running buffer 1:32 and in gel loading buffer (1:4) before gel loading. After the total protein dosage by the method of Bradford (Bio-Rad Protein Assay, Biorad, Herts, UK), an equal amount of sample volumes containing 50 µg of proteins were heat denatured for 5 min in Laemmli’s buffer. Then, 4–15% gradient polyacrylamide gels (Mini-PROTEAN TGX System, Bio-Rad, Herts, UK) were used for the electrophoretic separation of proteins. Separated proteins were finally transferred by wet electro-transfer onto nitrocellulose membranes (protran premium 0.45 uM, Amersham, UK). The protein-loaded membranes were then stained with Ponceau red S Staining Solution (ThermoFisher, Waltham, MA, USA) to confirm equal loading and correct transfer of protein. In addition, measuring the total amount of protein loaded/transferred in each lane provided data to be used for results normalization. After washing, the membranes were incubated for two hours at room temperature in Blocking Buffer (TBS-T/5% *w*/*v* nonfat dry milk) to saturate the non-specific sites. After three times washing for 5 min in washing buffer (TBS-T: 20 mM Tris-HCl, 150 mM NaCl, pH 7.4, 0.05% Tween20), the membranes were incubated overnight at 4 °C with the various primary antibodies resuspended in blocking buffer (dilution 1:1000). After incubation, the membranes were washed as above and subsequently incubated with secondary antibodies conjugated with alkaline phosphatase resuspended in blocking buffer for one hour. After washing, the specific proteins were revealed by a chemiluminescent assay (Pierce, ThermoFisher, Waltham, MA, USA). The membranes were covered with a thin layer of chemiluminescent alkaline phosphatase substrate and bands were visualized and analyzed by the ChemiDoc imaging system and software (Biorad, Herts, UK). The relative changes in serum protein levels of BDNF, NGF, VEGF, IGF-1, ANG, and END-1 after treatment were calculated as percentages vs. control/untreated samples.

In the case of barely visible bands on Western blots, the biochemical analyses were performed by Enzyme-Linked Immunosorbent Assay (ELISA) measurements following the manufacturer’s instructions (Human ELISA Kit; BT LAB, Birmingham, UK). Briefly, after thawing, serum samples were diluted at a ratio of 1:100 with PBS and added to the plate pre-coated with human-specific antibodies. After two hours at RT, a biotinylated human-specific antibody was added to reveal the specific neurotrophin captured on the plate from the samples. This step was followed by the classical incubation with HRP-conjugated streptavidin, which binds to the biotinylated primary antibody. HRP substrate addition for 10 min allowed the color development (blue-to-yellow change) proportional to the amount of specific neurotrophin. Each step was followed by accurate washing to eliminate the unbound molecules. After stopping the reaction with an acidic stop solution, the densitometric analyses of samples were obtained by measuring the absorbance at 450 nm, directly on the plate, using a microplate reader (Multiscan, Ascent, ThermoFisher, Waltham, MA, USA).

### 2.4. Transcranial Doppler Sonography

TCD is a non-invasive sonographic technique using a probe transducer ≤2 MHz to insonate the main basal arteries of the brain through a few specific thin windows of the skull. As such, TCD can probe in vivo the CBF velocities and resistances with a high temporal resolution and for a sustained time [61,62]. TCD has been recently employed to assess the occurrence and degree of small vessel pathology and related cognitive disorders in older individuals at risk of stroke [63] or GD [64,65].

An expert sonographer (R.B) performed TCD through a Compumedics DWL, Multi-Dop R X digital (2016, Singen, Germany). The middle cerebral artery (MCA) was insonated at a median depth of 56.0 mm, bilaterally. CBF velocity values from the MCA proximal tract were calculated from the transtemporal bone window employing a 2 MHz pulsed-wave Doppler ultrasound handheld probe. Each measurement was registered both at rest and at the depth resulting in the best signal (i.e., 50–60 mm). Indexes of interest were: peak systolic velocity (PSV), end-diastolic velocity (EDV), mean blood flow velocity (MV), pulsatility index (PI) (which was equal to: (PSV–EDV)/MBFV)), and resistivity index (RI) (according to the formula: (PSV–EDV)/PSV)) [66]. These parameters were obtained for at least 10 cardiac cycles and after a stable recording time of 30 [67].

**Figure 1:**
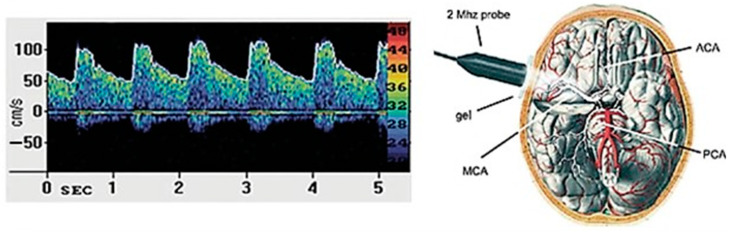
Schematic illustration of a standard TCD exam and main insonated arteries. ACA: anterior cerebral artery; MCA: middle cerebral artery; PCA: posterior cerebral artery.

A schematic illustration of a standard TCD exam and the main arteries insonated is presented in Figure 1.

### 2.5. Transcranial Magnetic Stimulation

MEPs were recorded bilaterally from the First Dorsal Interosseous (FDI) muscle using a 70 mm “figure-of-eight” coil connected to a Magstim 200 stimulator (The Magstim Company, Whitland, Dyfed, UK). Motor traces were filtered (bandwidth 3–3000 Hz) and amplified through a Medelec Synergy system (Oxford Instruments, Abingdon, UK), with a time sweep of 5 ms/div and a gain of 1 mV/div. Electromyographic (EMG) discharges were recorded through silver/silver-chloride self-conductive and self-adhesive disposable surface electrodes. The active was placed on the target muscle (FDI) muscular belly and the reference at the index finger metacarpal-phalangeal joint, whereas the ground electrode was on the dorsal wrist surface. For the motor nerve conduction study (M and F-waves), a bipolar nerve stimulation electrode with an interelectrode separation of 25-mm and 6-mm diameter felt pads was applied bilaterally to the ulnar nerve at the wrist.

Based on the latest guidelines of the International Federation of Clinical Neurophysiology [30], the resting motor threshold (rMT) was determined as the minimum intensity stimulation capable of eliciting MEPs with an amplitude >50 μV in at least 5 of 10 trials at rest. The rMT is viewed as an aggregate measure of cortical excitability, since it is a compound excitation index of the cortical motor neuron membranes, the neural inputs towards pyramidal cells, the spinal motor neurons, and the neuromuscular junctions, till the muscles.

Central motor conduction time (CMCT) was obtained by subtracting the conduction time along the peripheral nerves (according to the F-wave), from MEP latency elicited during active moderate muscular contraction, with an intensity of the stimulus of 130% of the subject’s rMT [30]. M- and F-waves were calculated through supramaximal electrical stimulation to the ulnar nerve at the wrist. MEP amplitudes were used as a percentage of the supramaximal M wave amplitude, i.e., the amplitude ratio (A ratio). MEP amplitude represents a compound measure of the excitation state of output cells within the motor cortex, the conduction along the peripheral motor nerve to the muscle [30].

The cortical silent period (cSP) was defined with approximately 50% of the maximal voluntary tonic contraction of FDI muscles, produced by single TMS pulses at 120% of the individual’s rMT. The mean cSP duration of 10 rectified trials was obtained. This index is thought to functionally measure the inhibitory intracortical circuitries, especially mediated by gamma-amino-butyric acid (GABA)-B activity [68,69]. The ipsilateral silent period (iSP) is elicited through TMS applied to the same hemisphere of a tonically contracted muscle, thus likely reflecting the transcallosal (GABAergic) inhibition [70]. As for cSP, both onset and duration are mainly considered for iSP, with longer duration seen as a stronger interhemispheric inhibition, and vice versa [71]. However, unlike cSP, iSP is hypothesized to be a totally cortical phenomenon: accordingly, it does not reduce the amplitude of the H-reflex, thus supporting the concept of a lack of spinal contributions [70].

All recordings were performed with patients comfortably seated on a dedicated chair, with constant EMG monitoring to ensure complete relaxation at rest or a continuous level of EMG activation during contraction. All data were collected on the Lab PC and safely stored through an ad hoc software for offline analyses [72].

**Figure 2:**
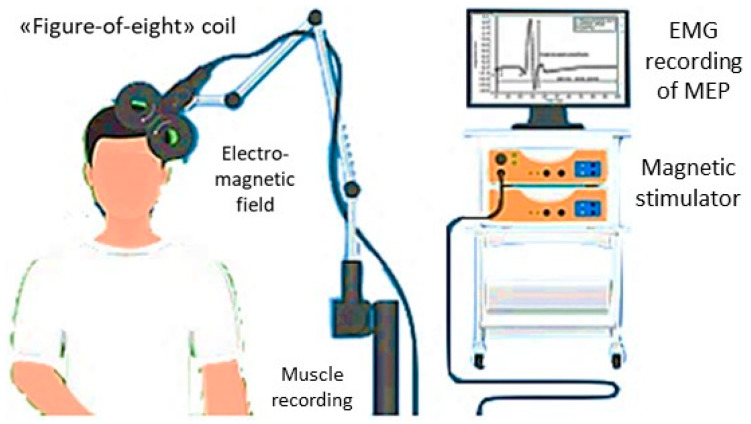
Basic principles and running of a standard TMS exam; an example of MEP in the screen. EMG: electromyiographic; MEP: motor evoked potential.

Figure 2 shows the basic principles and the running of a standard TMS exam.

### 2.6. Repetitive Transcranial Magnetic Stimulation

The rTMS was performed with a Magstim Superapid stimulator (The Magstim Company, Whitland, UK), connected to a 70-mm figure-of-eight coil. Overall, rTMS is a safe procedure, although, in a small percentage of cases, side effects may occur, such as headache, feeling unwell, and, very rarely, seizures. All these adverse events are reversible but, if they appear, they need to be reported.

The stimulation protocol consisted of a total of 15 sessions of high-frequency (10 Hz) rTMS over three weeks (five sessions per week, from Monday to Friday), at the same time of the day (approximately 11:00 a.m.), by the same trained operators (L.M. and G.S.). According to previous studies in patients with MD [44,46,73] and vascular depression [74], the optimal target site is the left DLPFC, which was identified by moving the coil 5 cm anteriorly in a parasagittal plane from the “hot spot” for the right FDI muscle to the scalp [75]. Each session consisted of a total of 3000 stimuli given in 4-second trains, with an inter-train interval of 26 s, for a total of 37.5 min per session. The stimulus intensity was set to a value equal to 120% of the individual’s rMT of the hemisphere corresponding to the stimulated DLPFC. According to the Clinical TMS Society consensus review and treatment recommendations for therapy of MD, daily left prefrontal TMS has substantial evidence of safety and efficacy for treating the acute phase of depression in patients who have an inadequate response or are intolerant to drug treatment [76].

**Figure 3:**
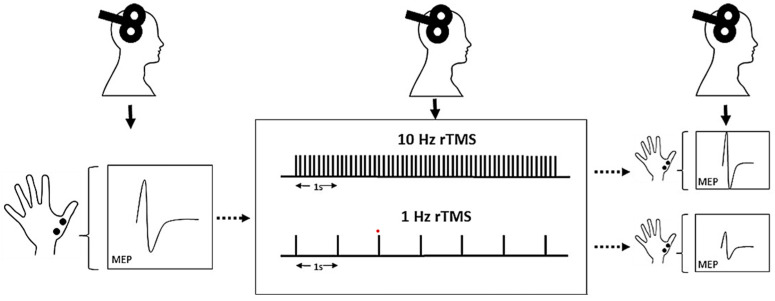
Basic protocols and main effects induced by rTMS. MEP: motor evoked potential. Modified from [77]. MEP: motor evoked potential; rTMS: repetitive transcranial magnetic stimulation.

Figure 3 illustrates the basic protocols and main effects induced by rTMS.

### 2.7. Statistical Analysis

Subjects’ characteristics were handled as median and IQR. Because of the small number of participants, all analyses were carried out by comparing the results obtained at T0 and T1 in each subject by mean on the non-parametric test of Wilcoxon for paired datasets. *p* value was considered statistically significant when <0.05.

## 3. Results

### 3.1. Descriptive Data

Demographic and clinical features of participants, along with their pharmacological regimens, are shown in Table 1. All patients were on one or more psychoactive drugs belonging to the class of antidepressants (tricyclics, selective serotonin reuptake inhibitors, serotonin, and norepinephrine reuptake inhibitors), antipsychotics (mostly of the new generation), and anxiolytics (benzodiazepines). None had relevant comorbidities, except for one or more vascular risk factors in five patients. Both general and neurological examinations were normal in all subjects, except for the presence of archaic reflexes in two patients and mild diffuse signs of parkinsonism (likely iatrogenic) in the other two.

Brain MRI was normal in the female patient, whereas in the remaining subjects, it showed signs of chronic subcortical ischemic vascular disease. In particular, according to the Fazekas visual scale, it was of moderate degree in two subjects (multiple chronic hypoxic-ischemic peri- and supraventricular WMLs, tending to confluence) and of mild severity in the other two (punctuate, not confluent, chronic hypoxic-ischemic WMLs).

**Figure 4:**
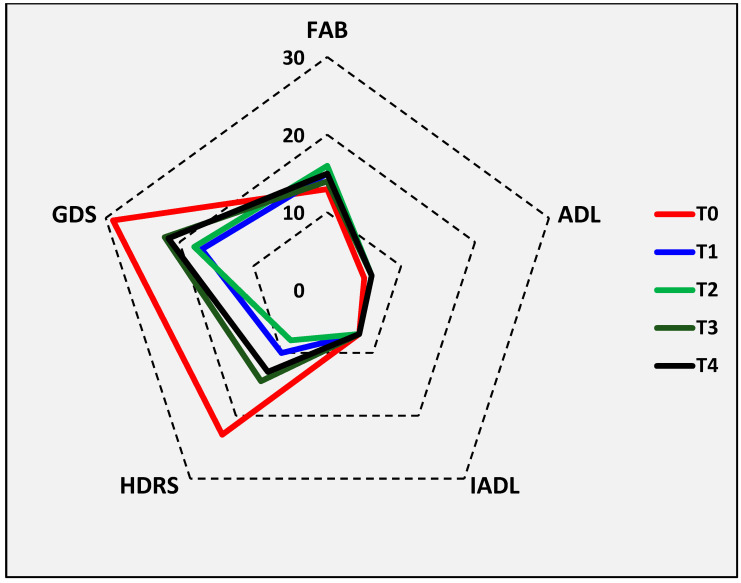
Graphic representation of the main neuropsychological test scores of all participants at baseline (T0) and at follow-up visits (T1–T4). ADL: Activities of Daily Living; FAB: Frontal Assessement Battery; GDS: 30-item Geriatric Depression Scale; IADL: Instrumental Activities of Daily Living; HDRS: 21-item Hamilton Depression Rating Scale.

No adverse or undesirable effect was reported during or after rTMS procedures. Compared to T0, statistically significant subjective improvement in mood was observed at T1 and T2 for HDRS only (whose median score decreased from 23.0 to 10.0 at T1 and to 8.0 at T2; both *p* = 0.028), whereas the other psycho-cognitive and functional scales did not change significantly at any of the follow-up visits, although GDS also showed tendentially lower scores at the same follow-up visits (Table 2 and Figure 4). All TMS measures remained basically unchanged after stimulation, except for a significant increase in the right median MEP amplitude (from 3.2 to 4.1 mV; *p* = 0.046) (Table 3). At TCD, although there was a bilateral increase in CBF, none of the TCD parameters varied significantly (Table 4).

**Figure 5:**
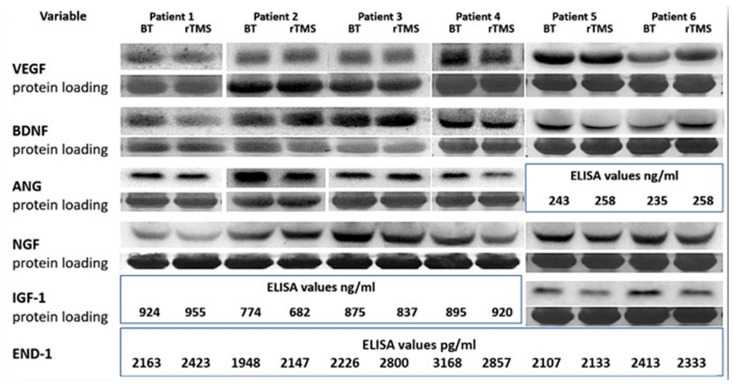
Representative Western blot images and ELISA measurements showing changes of selected growth factors compared to the samples before treatment. ANG: angiogenin; BDNF: brain-derived neurotrophic factor; BT: before treatment; ELISA: enzyme-linked immunosorbent assay; END-1: endothelin-1; IGF-1: insulin-like growth factor-1; NGF: nerve growth factor; rTMS: after treatment with repetitive transcranial magnetic stimulation; VEGF: vascular endothelial growth factor.

Finally, serum analyses showed considerable variability in serum changes in the neurotrophins considered. Indeed, after rTMS, different positive or negative fluctuations were observed, which ranged from −30% to 42% of specific neurotrophins in single patients (Table 5 and Figure 5).

### 3.2. General Considerations

To date, this is the first study exploring the effect of rTMS in GD patients through several objective measures derived from clinical, laboratory, and instrumental exams.

Taken together, we were unable to confirm the hypothesis of the study, i.e., a multimodal effect of rTMS in these patients with GD. However, notwithstanding the pilot design of the study and the small sample size, some results seem to emerge, in addition to the fact that rTMS was safe and well-tolerated in these patients. Although a transient positive effect on HDRS, high-frequency rTMS over the left DLPFC of these GD patients was unable to exert any relevant or persistent benefit on mood and cognition. This was in line with the absence of a remarkable effect on the laboratory and instrumental pre/post-rTMS measures considered here, i.e., levels of neurotrophic and growth factors, TMS metrics of cortical excitability, and TCD-based indexes of CBF, thus challenging, at least at this stage and in this sample, the concept of pleiotropic actions of rTMS. The presence of subcortical cerebrovascular disease and the exposure to multiple pharmacological treatments might have possibly contributed to the lack of effects in these patients.

From the neurosonological perspective (TCD), all the values recorded from the MCA, bilaterally, (PSV, EDV, MV) tended to increase after rTMS, suggesting a bilateral rTMS-induced modulation of CBF velocities along the main intracranial artery (which, as known, perfuses the anterior 2/3 of the brain, including the prefrontal regions). It should be noted that the velocimetric variations were found not only in the stimulated hemisphere but also contralaterally, thus hypothesizing both an ipsilateral and contralateral effect of rTMS on the cerebral hemodynamics of these patients. However, none of these measures reached a statistically significant difference. It should be noted that, in healthy subjects, 10 Hz-rTMS induced only a mild increase (3.6%) of CBF in the MCA on the stimulated hemisphere only, whereas the PI remained unchanged. It is likely that the increase of CBF is due to the dilatation of the small resistance vessels rather than due to vasoconstriction of the MCA [78]. This might occur even more in patients with GD, whose autonomic control of cerebral hemodynamics is altered [79].

At TMS and peripheral neurophysiological level, a similar lack of effect of rTMS was found on both cerebral hemispheres and ulnar nerves, except for a significant increase in the right median MEP amplitude, as expected after high-frequency rTMS [80]. However, this finding was not associated with any other change of TMS indexes of excitability and plasticity, including the more reliable A ratio, and therefore, the possibility of a stochastic effect cannot be excluded. However, more importantly, since cortico-motor plasticity is considered as a predictor of the response to high-frequency rTMS treatment for MD [81], the lack of effect we observed might indicate an impairment of the TMS-explored mechanisms of cortical excitability and synaptic plasticity in GD. This is also consistent with prior findings showing that MD patients tend to exhibit a blunted response (e.g., a reduced MEP facilitation) to plasticity-inducing protocols [82,83,84].

Biochemically, the patients recruited in this study were tested for the serum level of some neurotrophic and growth factors known to play a major role in the nervous system physiology and several neuropsychiatric disorders. However, conflicting results, as also observed elsewhere, emerged from our evaluation. Apart from the possibility of different individual responsiveness, it is reasonable to hypothesize that this variability might be due, at least in part, to the overlapping effects of multiple pharmacotherapies [85,86]. As such, any correlation of these changes with clinical outcomes after rTMS represents a challenge that requires further replication and systematic studies in larger samples.

## 4. Discussion

### 4.1. Proposed Pathomechanisms

The current model of depression conceptualizes it as a network disorder linked to diffuse changes in different brain regions. The left DLPFC and subgenual ACC were repeatedly associated with symptoms of depression [87,88]. Namely, the ACC is overactive in depression and a decreased activity correlates with an antidepressant response. Conversely, the left DLPFC is hypoactive in depression and the increased activity, as that induced by excitatory (high-frequency) rTMS (as in this study) is related to an antidepressant response [89]. However, the pathophysiology underlying GD differs from that of young patients with depression for several reasons. For example, some aging-related processes, such as vascular disease, amyloid accumulation, and inflammation, occur more frequently in the elderly, thus promoting dysfunctional frontal-subcortical networks, facilitating the onset of depression, and facilitating its recurrence and chronicity [90]. Additional factors seem to contribute, such as diabetes, obesity, hypertension, peripheral or carotid vascular disease (as in these patients), as well as an impaired synaptogenesis and neuroplasticity, which would start early in the in mid-life but would aggravate during aging [91]. Each of these stressors seems to trigger maladaptive responses, eventually leading to cerebral network diseases and different neuropsychiatric disorders [92].

Functional and structural brain network changes, especially secondary to age-related vascular injuries, were also demonstrated in GD. Specifically, diffusion tensor imaging reports in GD demonstrated microstructural changes in white matter tracts connecting the prefrontal cortex with the posterior cortical and subcortical areas, which have been related to executive dysfunction [93,94]. These functional and structural alterations seem also to be the correlate of apathy, psychomotor retardation, lack of insight, anhedonia, and insufficient response to antidepressant treatments, as noted in the patients enrolled in the present study and as clearly shown in previous investigations [95].

Interestingly, these features might represent the clinical correlates of the lack of rTMS effects observed in this study. In this context, it should be acknowledged that, although some undoubted advantages of TMS in the elderly, such as less undesired events with respect to antidepressant drugs and the lack of adverse cognitive effects compared to the electroconvulsive therapy, only half of the subjects with drug-resistant MD reach an adequate symptom control, with 25–30% only attaining remission [96]. Several attempts have been recently carried out to refine the rTMS setup (e.g., targeting, timing, dosage) to improve its antidepressant effects, albeit they are still based on the brain model of younger patients, thus questioning the feasibility in GD, also based on the different brain anatomy and neurochemistry of older subjects [89]. For example, brain atrophy may increase the distance between brain tissue and the stimulating coil; similarly, the increased content of cerebral spinal fluid might affect the propagation of the current produced by TMS [97]. As such, further effort is still needed to optimize TMS so that it can be used systematically in GD.

Nevertheless, it has been argued that older individuals might be less likely to respond to TMS for depression, thus making GD a less favorable target for neuromodulatory interventions. For this reason, in 2015 Sabesan et al. considered the factors that can moderate the clinical effect of TMS in GD [98], i.e., the brain atrophy, the intensity and number of pulses (or, in other words, the dose-response relationship), and the patients’ clinical profile. In 2018, Iriarte and George further reviewed the factors that influence the response to TMS in elderly individuals [99], whereas in 2020, van Rooij et al. discussed the influence of the aging brain on rTMS efficacy for GD and highlighted the importance of developing specialized rTMS protocols for treating depression in the elderly [100]. Lastly, a very recent systematic review included seven randomized controlled trials and seven uncontrolled trials [89]. Overall, the authors found substantial variability in the clinical response, ranging from 6.7% to 54.3%. The literature reviewed also showed large heterogeneity among studies, both for patients included and TMS protocols adopted [89].

However, in our study, it is unlikely that the lack of clinical response could be attributed to the technical factors or the methodological issues previously reviewed. First, although the cortex-scalp distance was not estimated, none of the patients had brain atrophy at MRI and all of them underwent suprathreshold rTMS (at 120% rMT), as recommended [74]. Conversely, Manes et al. (2001) [101] stimulated the left DLPFC at 80% rMT and did not find a difference in either response or remission rate between the active and the sham group, whereas Jorge et al. (2008) [74] stimulated the same cortical region at 110% rMT and found a significant difference in both outcomes.

Second, the total pulse count affects the clinical outcome, especially for older adults, given the evidence of altered, hypoactive, mechanisms of plasticity with advancing age [102,103]. Although a total number of stimuli currently has not been recommended, Jorge et al. [74] carried out two trials: in the first experiment, the patients received a total of 12,000 pulses throughout the intervention, whereas in the second one, the total amount was 18,000. The researchers noted a higher response (39.4% vs. 33.3%) and remission rate (27.3% vs. 13.3%) with 18,000 total pulses. In this study, a total of 45,000 stimuli were delivered. Nevertheless, we could not observe any substantial, long-lasting, clinical response.

Third, so far, no study has systematically investigated how ischemic WMLs affect TMS-induced plasticity. Each of these brain-related modifications may influence how the TMS magnetic field reaches the cortex and alters the response to treatment, thus possibly explaining, at least in part, the impairment of modulation of electrocortical microcircuits to TMS, the level of neurotransmitter activities, and the changes of CBF to TCD. Therefore, although the neurotrophins studied here might be involved in the antidepressant effect of rTMS [104,105], the suitability of each as a disease marker and outcome measure in GD requires further understanding and larger samples. This is even more important, considering that this reduction in functional connectivity correlates with age-related cognitive deterioration [106], WMLs [107], and decreased connectivity by aging [108]. In this regard, Opie et al. (2017) [109] demonstrated that intermittent theta burst stimulation increased M1 plasticity in the young but not in the older group [109].

Biochemically, several neurotrophins and growth factors are involved in the pathogenesis of mental illnesses and might be used as biomarkers of cognitive, behavioral, or emotional deficits [110]. Among others, some alterations in gene expression and/or serum levels of specific growth factors have been associated with the development and/or course of depression [111], whereas the molecular pathways underlying the rTMS effect and predicting the response to this treatment remain to be elucidated. Therefore, the identification of specific biochemical events involved in the consistent effect of rTMS in neurological and psychiatric disorders may also provide diagnostic and prognostic hints [110,112].

However, although it is not possible to draw conclusions regarding the variations of the neurotrophins and growth factors measured here, the potential of rTMS in modulating their serum concentration remains crucial. For instance, BDNF is a key factor in the development and treatment of depression, though studies of the effects of rTMS on BDNF in patients with MD have shown conflicting results. The rTMS studies to date available in humans showed that plasma levels of BDNF in MD patients significantly increased after treatment in responders and partial responders, but not in non-responders [113,114,115], which may be the case of the present study. In addition, there is also a different impact of BDNF genotypes on the effect of left-sided prefrontal stimulations [116].

Regarding VEGF, it has been demonstrated that individuals successfully treated with rTMS had a significant increase in VEGF after treatment compared to the baseline and the non-responders/non-remitters. Notably, the greater increase in VEGF was related to higher improvement in depressive symptomatology after TMS [117]. This provides correlative data that encourage future investigations exploring VEGF as both a key mediator in the process underlying the antidepressant effect of TMS and a possible marker of clinical outcome. Nevertheless, another two recent studies found no significant association between depressive scores and serum VEGF levels at any timepoint after rTMS [115,118], which may be also the case of the present investigation. Moreover, the level of VEGF was significantly higher in non-responders than in responders and baseline VEGF significantly predicted the response to treatment [118].

Finally, although ANG has been shown to be related to depressive symptoms, the conflicting percentage variation observed in the present study after rTMS did not allow to draw conclusions. The same holds true for NGF, another important member of neurotrophin, which is dysregulated in the pathophysiology of depression. However, to our knowledge, there is no previous TMS study on it and even the evidence of the effect of antidepressant drugs on modulating depression via the NGF both preclinical and clinical models of depression are conflicting [119]. Similarly, the intake of antidepressant drugs changed the association between plasma IGF-I levels and anxiety/depressive disorders. Higher levels of IGF-I may reflect an adaptive mechanism to counterbalance the impaired neurogenesis, though further investigation should support this concept [120]. Regarding END-1, it decreased the diabetes-induced inflammation in the hippocampus, thus postulating a neuroprotective action. Given that endothelial cells and neurons in the neurovascular unit depend on each other for functioning properly, it has been recently demonstrated that the blockage of END-1 might protect the hippocampus through the modulation of the BDNF system [121]. However, data on its involvement in GD are currently lacking.

Lastly, as above-mentioned, psychotropic medications may alter cortical excitability and synaptic plasticity, thus exerting differential influence on clinical response to rTMS and outcome measurements in MD patients [122]. In this study, patients were chronically treated with antidepressants, neuroleptics, and benzodiazepines. However, TMS literature on patients on chronic drug intake is rather few. In summary, benzodiazepines have moderate and variable effects on plasticity and some effects on excitability [123]. For instance, lorazepam has no effects on rMT and/or active MT [124], whereas it may clearly alter CSP and MEPs at rest [125]. Of note, response rates to rTMS and clinical improvement were found to be lower in benzodiazepine users versus non-users, since chronic intake seems to mitigate the increases in cortical GABAergic signaling that emerges with clinically effective rTMS [126]. Accordingly, in a recent observational study, the improvement of depressive symptoms was significantly less pronounced in patients taking lorazepam [127]. Regarding antidepressants, those with potent serotonin transporter inhibition (such as citalopram) seem to reduce cortical excitability by increasing the rMT and impair plasticity [123,128], both after intravenous and oral administration [129]. Finally, quetiapine can cause CSP prolongation after even a single dose, without affecting MT, intracortical inhibition and intracortical facilitation [130]. Remarkably, changes in CSP can impact the cortical “receptivity” for rTMS and impair long-term neuroplastic mechanisms [131,132]. In this context, some authors [133] found that blocking dopamine receptors by antipsychotic drugs might attenuate the positive effects of rTMS, although others [134] observed that outcomes were not significantly affected. Overall, more studies are needed to conclude whether psychoactive drugs on cortical excitability and synaptic plasticity are associated with greater or lower improvement after rTMS.

### 4.2. Limitations

The main limitations of this study are the very small sample size, the prevalence of male participants, and the potential interference of psychoactive drugs on the outcome measures, especially on TMS metrics and laboratory measurements.

In addition, the lack of sham (fictious) stimulation did not allow to exclude a placebo or learning effect potentially explaining the effect on HDRS, although the use of objective outcome measures (such as those derived from TMS and TCD) may have mitigated this scenario. The same holds true for the lack of control sample without depression, i.e., non-depressed individuals with signs of chronic subcortical ischemic vascular disease, to determine whether their neurotrophic/growth factor differed from those of patients at T0.

Moreover, it cannot be excluded that the effect observed in HDRS scores may be due, at least in part, to lower statistical power of GDS compared with HDRS [135]. Additionally, the use of different study models, in particular, in different models of patients and grouping, also considering the drugs taken, can help in disentangle these complex multiple interactions.

Finally, the lack of a clear evidence makes this study basically descriptive. Further investigations on larger samples are needed to acknowledge the relevance of properly dosing TMS protocols for GD and adapting interventions and parameters to older adults.

## 5. Conclusions

In this case series study, we were unable to confirm the hypothesis of the study, i.e., a multimodal effect of rTMS in GD. Both cerebrovascular disease and chronic exposure to multiple pharmacological treatments might have contributed. However, the lack of consistent clinical response suggests that the treatment might be potentially optimized.

## Figures and Tables

**Table 1:** Participants’ demographic and clinical features, along with the pharmacological regimen.

Variable	Patient 1	Patient 2	Patient 3	Patient 4	Patient 5	Patient 6
Sex	Female	Male	Male	Male	Male	Male
Age, years	62	81	82	60	72	64
Education, years	18	5	3	13	8	13
Height, cm	160	165	170	170	168	177
Weight, Kg	57	62	70	63	72	72
Family history of depression	No	Yes	No	No	No	Yes
Current episode duration, months	9	6	10	7	5	24
Vascular risk factors	None	Dyslipidemia Diabetes	Hypertension Dyslipidemia Mild bilateral carotid stenosis	Tobacco smoking	Left carotid stenosis	Hypertension
Brain MRI	Normal	Mild SCVD	Moderate SCVD	Mild SCVD	Moderate SCVD	Mild SCVD
Drug daily dosage(s)	cital 40 mg olanz 5 mg loraz 2.5 mg venla 75 mg	vorti 5 mg	vorti 5 mg queti 25 mg	amitr 50 mg queti 350 mg loraz 5 mg	aripi 5 mg cital 20 mg	diaze 2.5 mg queti 50 mg cital 40 mg amitr 60 mg triaz 0.5 mg

amitr = amitryptiline; aripi = aripiprazole; cital = citalopram; diaze = diazepam; loraz = lorazepam; MRI = magnetic resonance imaging; olanz = olanzapine; queti = quetiapine; SCVD = subcortical vascular disease; triaz = triazolam; venla = venlafaxine; vorti = vortioxetine.

**Table 2:** Neuropsychological test scores of all participants at baseline (T0) and at follow-up (T1).

Variable	Median (IQR) T0	Median (IQR) T1	Wilcoxon Z	*p* Value
MMSE	27.7 (24.0–29.0)	28.2 (17.5–30.0)	0.405	0.686
FAB	12.0 (10.0–17.0)	15.0 (10.0–18.0)	1.483	0.138
Stroop T	27.6 (22.4–57.7)	32.3 (28.7–40.0)	0.314	0.753
Stroop E	2.0 (1.0–3.0)	1.5 (0.0–3.0)	0.135	0.893
Stroop score	15.7 (7.3–42.8)	22.0 (9.1–26.1)	0.314	0.753
ADL	5.5 (5.0–6.0)	6.0 (6.0–6.0)	1.342	0.180
IADL	5.0 (4.0–7.0)	7.0 (5.0–7.0)	1.826	0.068
HDRS	23.0 (19.0–24.0)	10.0 (8.0–16.0)	2.201	**0.028**
GDS	22.5 (22.0–26.0)	17.0 (16.0–27.0)	1.363	0.173
AES	43.0 (40.0–49.0)	43.0 (39.0–45.0)	0.405	0.686

IQR = interquartile range; MMSE = Mental State Examination; FAB = Frontal Assessment Battery; Stroop T = Stroop Color-Word Interference Test, time (seconds); Stroop E = Stroop Color-Word Interference Test, number of errors; Stroop score = Stroop Color-Word Interference Test, interference score; ADL = Activities of Daily Living; IADL = Instrumental Activities of Daily Living; HDRS = 21-item Hamilton Depression Rating Scale; GDS = 30-item Geriatric Depression Scale; AES = Apathy Evaluation Scale; numbers in bold = statistically significant *p* value.

**Table 3:** Electrophysiological data to TMS of all participants at baseline (T0) and at follow-up (T1).

Variable	Median (IQR) T0	Median (IQR) T1	Wilcoxon Z	*p* Value
Left rMT, %	40.5 (39.0–42.0)	40.0 (38.0–41.0)	1.348	0.178
Left MEP latency, ms	22.0 (20.0–22.5)	21.7 (20.8–22.0)	0.405	0.686
Left MEP amplitude, mV	4.1 (3.7–5.3)	4.7 (3.9–7.0)	1.153	0.249
Left PMS latency, ms	15.5 (15.1–16.4)	16.0 (14.1–16.2)	0.524	0.600
Left CMCT, ms	6.4 (6.1–6.9)	6.2 (5.4–7.2)	0.105	0.917
Left CMAP latency, ms	3.2 (2.7–3.9)	3.1 (2.8–3.4)	0.730	0.465
Left CMAP amplitude, mV	17.4 (10.3–22.2)	18.0 (17.2–20.6)	0.734	0.463
Left F wave latency, ms	30.7 (29.1–31.8)	31.0 (29.4–31.8)	0.405	0.686
Left F wave amplitude, mV	0.2 (0.1–0.2)	0.2 (0.1–0.2)	0.943	0.345
Left amplitude ratio	0.3 (0.2–0.4)	0.3 (0.2–0.4)	0.314	0.753
Left CMCT-F, ms	5.3 (5.0–5.5)	5.6 (4.4–6.1)	0.629	0.529
Left cSP, ms	70.6 (59.8–94.0)	67.2 (48.8–78.0)	0.734	0.463
Left iSP, ms	17.8 (13.2–19.8)	16.5 (13.7–17.5)	0.524	0.600
Right rMT, %	39.5 (35.0–50.0)	39.0 (36.0–50.0)	0.000	1.000
Right MEP latency, ms	21.6 (20.7–21.9)	21.4 (20.1–22.2)	0.314	0.753
Right MEP amplitude, mV	3.2 (2.5–4.1)	4.1 (3.4–4.2)	1.992	**0.046**
Right PMS latency, ms	15.6 (14.5–16.0)	15.8 (14.9–16.1)	0.270	0.787
Right CMCT, ms	6.4 (5.5–6.6)	6.1 (5.2–6.3)	0.943	0.345
Right CMAP latency, ms	3.3 (2.8–3.7)	3.1 (2.7–3.6)	2.023	0.053
Right CMAP amplitude, mV	15.7 (14.2–16.8)	17.5 (12.5–21.0)	0.524	0.600
Right F wave latency, ms	29.7 (28.5–31)	30.1 (29.5–30.8)	0.524	0.600
Right F wave amplitude, mV	0.1 (0.1–0.2)	0.1 (0.1–0.2)	0.314	0.753
Right amplitude ratio	0.2 (0.2–0.3)	0.2 (0.2–0.4)	0.734	0.463
Right CMCT-F, ms	5.5 (4.9–5.8)	5.2 (5.0–6.1)	0.405	0.686
Right cSP, ms	77.8 (65.1–109.5)	72.1 (59.6–85.8)	0.943	0.345
Right iSP, ms	19.4 (15.7–20.0)	18.0 (17.2–28.9)	1.153	0.249

Legend: TMS = repetitive transcranial magnetic stimulation; IQR = interquartile range; rMT = resting motor threshold; MEP = motor evoked potential; PMS = peripheral motor stimulation; CMCT = central motor conduction time; CMAP = compound motor action potential; CMCT-F = central motor conduction time calculated with the F wave technique; cSP = contralateral cortical silent period; iSP = ipsilateral silent period; numbers in bold = statistically significant *p* value.

**Table 4:** Neurosonological features to transcranial Doppler sonography (TCD) of all participants at baseline (T0) and at follow-up (T1).

Variable	Median (IQR) T0	Median (IQR) T1	Wilcoxon Z	*p* Value
Left PSV, cm/s	62.5 (48.0–65.0)	75.5 (71.0–85.0)	1.153	0.249
Left EDV, cm/s	25.5 (18.0–28.0)	29.5 (20.0–42.0)	0.314	0.753
Left MV, cm/s	38.0 (35.0–42.0)	45.5 (39.0–61.0)	1.153	0.249
Left PI	0.9 (0.8–1.0)	1.0 (0.7–1.2)	1.363	0.173
Left RI	0.6 (0.6–0.6)	0.6 (0.5–0.7)	1.153	0.249
Right PSV, cm/s	58.5 (52.0–68.0)	71.0 (60.0–85.0)	0.674	0.500
Right EDV, cm/s	26.0 (20.0–31.0)	28.5 (24.0–42.0)	0.943	0.345
Right MV, cm/s	38.0 (32.0–44.0)	42.0 (36.0–61.0)	0.629	0.529
Right PI	0.9 (0.8–1.0)	0.9 (0.7–1.0)	1.214	0.225
Right RI	0.6 (0.5–0.6)	0.6 (0.5–0.6)	0.135	0.893

Legend: IQR = interquartile range; PSV = peak systolic velocity; EDV = end diastolic velocity; MV = mean velocity; PI = pulsatility index; RI = resistance index.

**Table 5:** Percentage variation of neurotrophic and growth factors compared to the baseline (T0).

Variable	Patient 1	Patient 2	Patient 3	Patient 4	Patient 5	Patient 6
VEGF	−13%	8%	−17%	−8%	−6%	42%
BDNF	−30%	2%	35%	−12%	−17%	31%
ANG	−10%	−20%	20%	−10%	5%	8%
NGF	−12%	−10%	−23%	−30%	−12%	−17%
IGF-1	4%	−12%	−5%	3%	−24%	−16%
END-1	12%	10%	25%	−20%	0%	0%

Legend: BDNF: brain-derived neurotrophic factor; VEGF: vascular endothelial growth factor; ANG: angiogenin; IGF-1: insulin-like growth factor-1; NGF: nerve growth factor; END-1: endothelin-1.

## Data Availability

All the relevant data for this study are contained within the article.

## Supplementary Materials

The following supporting information can be downloaded at: https://www.mdpi.com/article/10.3390/biomedicines11030958/s1, Table S1: CARE checklist.
